# Experiments to understand crystallization of levitated high temperature silicate melt droplets under low vacuum conditions

**DOI:** 10.1038/s41598-020-77965-4

**Published:** 2020-12-01

**Authors:** Biswajit Mishra, Pratikkumar Manvar, Kaushik Choudhury, S. Karagadde, Atul Srivastava

**Affiliations:** 1 Department of Mechanical Engineering, Indian Institute of Technology Bombay, Powai, Mumbai 400076 India; 2 Present Address: Iota Design & Innovations Lab Pvt. Ltd., CrAdLE, EDII, Gandhinagar, Gujarat 382428 India

**Keywords:** Engineering, Imaging techniques, Planetary science, Early solar system

## Abstract

We report experiments on crystallization of highly undercooled forsterite melt droplets under atmospheric and sub-atmospheric pressure conditions. Experiments have been conducted under non-contact conditions using the principles of aero-dynamic levitation. Real time dynamics of solidification, along with the transient evolution of surface textures, have been recorded using high speed camera for three cooling rates. These images have been matched with the time-tagged temperature data to understand the effect of pressure conditions and cooling rates on the crystallization dynamics. Compared to normal pressure, relatively higher levels of undercooling could be achieved under sub-atmospheric conditions. Results showed a strong dependence of surface textures on pressure conditions. For any externally employed cooling rate, relatively small length scale morphological textures were observed under sub-atmospheric conditions, in comparison to those achieved under ambient conditions. The observed trends have been explained on the basis of influence of pressure conditions on recalescence phenomenon and the rate at which latent heat of crystallization gets dissipated from the volume of the molten droplet. Sub-atmospheric experiments have also been performed to reproduce one of the classical chondrule textures, namely the rim + dendrite double structure. Possible formation conditions of this double structure have been discussed vis-à-vis those reported in the limited literature. To the best of our knowledge, the reported study is one of the first attempts to reproduce chondrules-like textures from highly undercooled forsterite melt droplets under sub-atmospheric non-contact conditions.

## Introduction

Forsterite, largely found in chondrites, has always been considered as one of the most primitive silicate material. It has been found in the cometary dust of the meteorites^1^ and believed to be present as tiny crystals in the dusty clouds of gas around a forming star^2^. Researchers seek to get an insight into the dynamics of evolution of materials, from the protoplanetary disk, into the present-day form, by understanding the nature of crystallization of the chondrites^3^. Apart from this, a conceptual understanding of the behaviour of materials formed from high temperature crystallization is of interest to semiconductor^4,5^ and glass-forming alloys^6,7^ sectors.

A wide range of models have been developed explaining the crystallization of chondrules in the early solar system^8–10^. One section of the astronomical society argues that crystallization took place under low cooling rates. Motivated by these studies, a considerable number of experiments have been performed under such conditions^11–13^ with an intent to reproduce some of the commonly encountered chondrule textures, for instance as reported in Ref.^14^. On the other hand, the other section believes crystallization took place under rapid cooling rates^15,16^. Using the principles of aero-acoustic levitation, Tsukamoto et al.^17^ attempted for the first time reproducing chondrule textures using container-less experimental conditions. Tangeman et al.^18^ used aero-acoustic levitation in the synthesis of vitreous forsterite. It was observed that slow cooling of the levitated forsterite melt lead to recalescence i.e. the release of latent heat of crystallization, which leads to an increase in the melt’s temperature^14^, while the rapid cooling lead to formation of glassy forsterite.

With the recent developments, researchers have made efforts into understanding the crystallization process by the use of some of the high-end scientific tools in-situ. In this direction, Aoyama and Kuribayashi^4^ used high speed camera to visualize and investigate the growth mechanism process of Si and Ge. Lü et al.^19^ used high speed camera to examine the transition of Ni–Zr alloy to its peritectic phase under different undercooling in an electromagnetic levitation system. Fukuyama et al*.*^20^ developed an ultrahigh-temperature thermal analyser, which was used in-conjunction with electromagnetic levitation to perform solidication and microstructural study of MoSiBTiC alloy. Srivastava et al.^21^ developed in-house optical setup for in situ visualization of convections inside the levitated forsterite melt before and during the crystallization process. Nagashio et al.^22^ performed real-time XRD experiments to understand the grain refinement dynamics involved during solidification of Si. These growth mechanisms have been observed to vary with levels of undercoolings for Si and Ge melts^23^. A numerical study was carried out by Miura et al.^24^ on the effect of supercooling and cooling rate on the growth mechanism for forsterite melts. In order to achieve different levels of undercooling, the melts were heterogeneously nucleated. Theoretical analysis of similar problems pertaining to solidification of semi-transparent melt particles for various applications have been reported^25–28^.

Although the container-less methods are best suited to achieve the objective of experimentally simulating the chondrule formation conditions that are as close as possible to those present in the early solar system, but the challenges associated with reproducing other realistic conditions have restricted the development of a clear understanding of the chondrule formation mechanism(s). The challenges include ensuring zero (or even microgravity) conditions, reproduction of exact gaseous environment around the solidifying sample etc. Several models investigating the formation mechanisms of chondrules under varying magnitudes of gravity have been reported^15,29^. Although the most likely scenario is the case of proper vacuum conditions with zero gravity, but the realization of both these conditions simultaneously under lab environment is almost impossible. With regard to the experiments conducted under microgravity conditions, some of the notable studies include the works reported by Liu et al.^30^ and Nagashima et al.^16^. Liu et al.^30^ studied the nucleation rate of CaCO_3_ from an aqueous solution under microgravity conditions and reported an increase in the nucleation rate by about four orders of magnitude vis-à-vis normal gravity conditions. Based on the experiments conducted using parabolic flights, Nagashima et al.^16^ found that nucleation of enstatite melts was delayed under microgravity conditions. However, while parabolic flights offer reduced gravity conditions, the molten sample needs to be held using a substrate or at most using a sample holding wire. The contact of the molten sample with the substrate and/or the sample holding wire leads to heterogeneous nucleation, which, in turn, restricts the levels up to which the molten droplet may be undercooled. Another major limitation of parabolic flight experiment lies in its inability to create reduced pressure conditions. In any experimental attempt made towards reproducing chondrule-like textures, it is important to ensure low pressure conditions (sub-atmospheric pressure) as it is widely believed that the naturally found chondrules crystallized under an environment that was characterized by extremely low pressures (vacuum) conditions. However, under lab-based conditions, performing completely container-less experiments and that too under vacuum conditions is highly challenging and probably, this is one of the reasons on the complete lack of literature that have reported any such kind of experiments focussing on the reproduction of chondrule-like textures.

Against this backdrop, the current study is aimed towards gaining an insight into the effect of pressure conditions (atmospheric and sub-atmospheric) on the growth mechanisms and the plausible morphological textures of the crystallized forsterite molten droplets. In order to ensure container-less conditions, aerodynamic levitation system was used in the experiments. The sub-atmospheric pressure condition was set at 50 mBar using a turbo-vacuum pump and a comparative study was conducted between the results obtained under atmospheric and sub-atmospheric conditions. In addition, the effect of cooling rate (100, 200 and 400 K/s) on the melt’s behaviour has also been investigated. The choice of the cooling rates employed has been motivated by some of the earlier studies available in the literature, for instance, Nagashima et al*.*^16^ wherein the authors suggested cooling rate of the order of 100 K/s in the context of chondrule crystallization. Real time evolution of surface textures during the crystallization of silicate melt droplets has been captured using a high-speed camera. Plausible effects of pressure conditions on solidification phenomena have been discussed in terms of the observed differences in the surface textures of the samples solidified under atmospheric and sub-atmospheric pressure conditions. The observations made in the experiments have been explained on the basis of the influence of pressure conditions on the recalescence phenomenon and the rate at which the latent heat of crystallization gets dissipated from the volume of the solidifying molten droplet. Experiments under sub-atmospheric conditions have also been performed to reproduce one of the classical chondrule textures, namely the double structure of rim + dendrite. The possible formation conditions of this double structure have been discussed vis-à-vis those reported through numerical simulations by Miura et al.^24^.

## Apparatus and instrumentation

Non-contact experiments conducted under atmospheric as well as sub-atmospheric conditions were carried using an aerodynamic High Temperature Conical Nozzle Levitator (HTCNL). Starting material of forsterite (Mg_2_SiO_4_) composition, in the form of a spherule (diameter ≈ 2 mm) was levitated on a gas jet and heated to temperature levels that were sufficiently high to convert the starting solid spherule into its molten form. Compared to the melting point temperature of forsterite (≈ 2163 K), the spherules were heated to the temperature levels of close to 2200–2473 K. This exercise ensured complete melting of the levitated samples before subjecting them to the predefined cooling rates under atmospheric and sub-atmospheric conditions. Choice of the forsterite material was based on the fact that the natural samples of chondrules have revealed the presence of forsterite (Mg_2_SiO_4_) in either crystalline or glassy form^31^. It is to be mentioned here that while the natural chondrule samples have also shown the presence of other elemental composition, for instance, ferrous, volatile elements such as Na, alkaline earth metals such as Ca etc., along with magnesium silicate, the present work employs samples of pure forsterite (Mg_2_SiO_4_) composition for the sake of simplicity and to avoid any plausible effects of other phenomena such as isotopic fractionation etc. Since the focus of experiments reported is to understand the plausible effects of reduced pressure conditions (vis-à-vis standard ambient pressure conditions) on the solidification of highly undercooled molten droplets and the resultant morphological textures reproduced under completely non-contact conditions, the choice of the composition of the starting material (Mg_2_SiO_4_) is reasonably justified.

**Figure 1:**
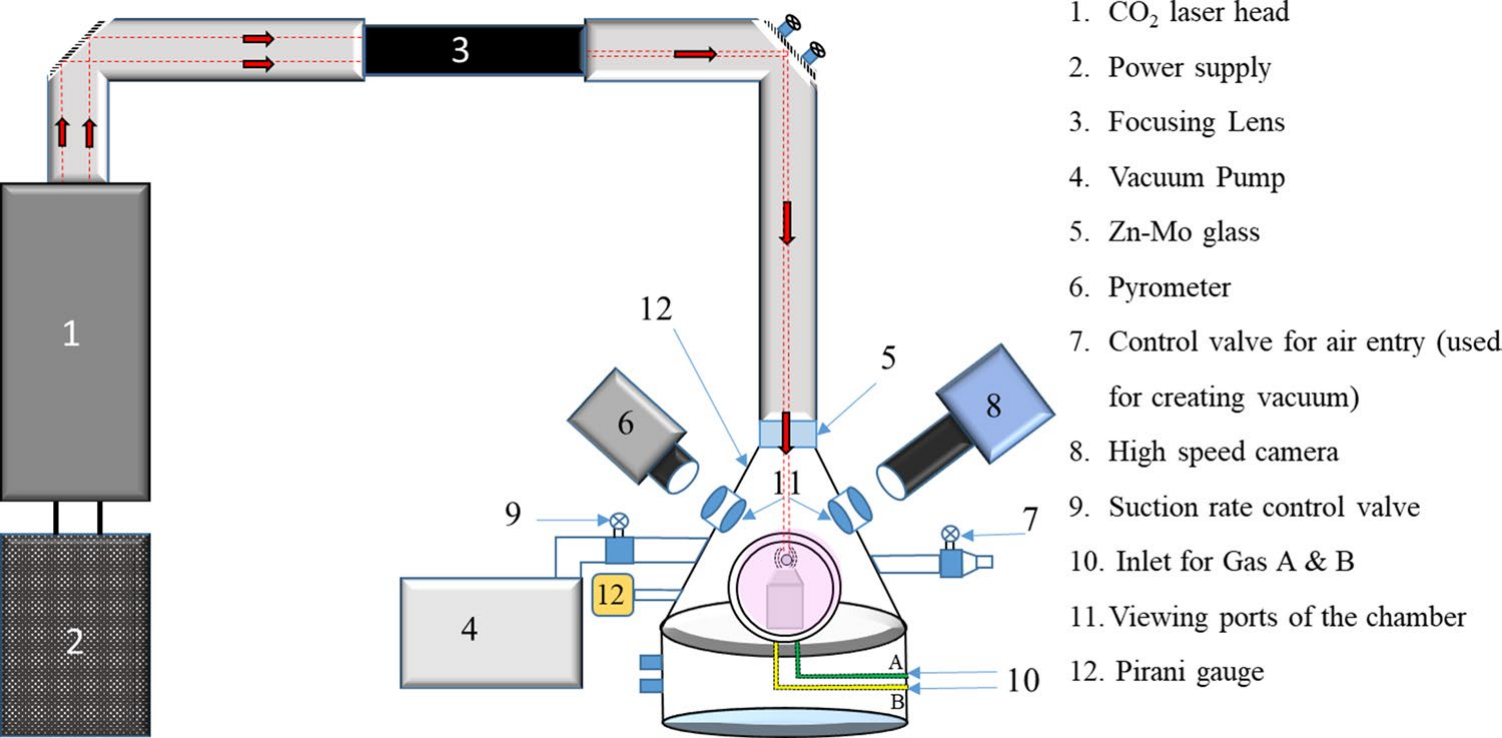
Schematic of the complete test rig for conducting non-contact solidification experiments using the principles of aero-dynamic levitation.

Figure 1 schematically shows the complete view of the levitation system on which the reported experiments have been conducted. Major components of the high temperature conical levitator (HTCNL) are levitation chamber fitted with glass windows, a high-power CO_2_ laser, vacuum pump, optical pyrometer and high-speed camera. The CO_2_ laser (*Firestar i400*, beam diameter: 6 mm ± 0.6 mm) along with a compatible controller is capable of delivering a maximum power of 400 W on the surface of the levitated sample with the help of a beam steering and focussing optics fitted with it. The CO_2_ laser emits at a wavelength of 10.6 µm. The laser beam has been guided through mirrors and lenses and hence is enclosed, using a tubing of anodized aluminium. Also, there are appropriate interlocks that can immediately switch off the laser radiation if any of the viewports are open in the levitation chamber.

As mentioned above, a high power CO_2_ laser (maximum power 400 W) has been employed in the experiments to raise the temperature of the levitated sample and convert it into its molten state. As part of experimental planning, a physical estimate of the total thermal energy required to heat and eventually melt the starting material has been made taking into account the various sub-processes associated with the phenomenon under study. These sub-processes majorly include the conversion of the initial solid spherule (at nearly room temperature) into its molten state (melting point temperature of forsterite ≈ 1890 °C), superheating of the molten sample up to a temperature of around 2200 °C (so as to ensure complete melting of the levitated sample). In addition, loss of thermal energy associated with radiative heat transfer and forced convective effects (due to the flow of Argon gas to levitate the sample) have also been accounted for. In these analyses, the thermos-physical and optical properties of forsterite, as documented in the works of Miura et al.^24^ and Lane et al.^32^, have been employed. Convective losses have been quantified using the standard correlation for Nusselt number for flow over a sphere^33^. Following this approach, the total thermal power required (including losses) for transforming the initially solid spherule into its complete molten state has been estimated to be ≈ 72 W. Considering some of the other unavoidable losses of laser power (arising out of factors such as optics-based beam steering unit, laser beam size being slightly larger than the cross-section of the spherule etc.), the net laser power that is deposited on the levitated sample has been kept sufficiently high so as to achieve the desired peak temperatures of around 2200 °C and to ensure complete melting of the starting material.

The levitation chamber is made up of stainless steel and has viewports (NW 25 and NW 40) for imaging the levitated sample and connecting vacuum lines. This chamber houses the conical nozzle and the base of the nozzle is connected to the gas inlet, through which the gas can be pumped to achieve aerodynamic levitation. Levitation has been achieved using Argon gas, which is inert to any chemical reaction with the material of the levitated sample. The entire levitation chamber, including, the gas inlet is maintained at 20* °C* by circulating chilled water. The laser temperature is maintained static at 20* °C* (so as to have a steady power output) using another chiller unit that takes away the heat and maintains the laser temperature.

Real time images of the crystallization phenomena (starting from the stage wherein the sample is in a complete molten stage to the end of crystallization) have been recorded using a high-speed CMOS camera (*Vision Research; Model: VEO 410L*). The camera offers a maximum frame rate of 5200 fps at full resolution of 1Megapixel. At such high speeds, the exposure times are very low and lead to poor image quality. To circumvent this limitation, an additional diffused light source was used to illuminate the sample. To conduct the experiments under sub-atmospheric conditions, a vacuum pump (Make: *Edwards*, dry scroll) was employed that has a peak pumping capacity of 11.4 m^3 ^h^−1^. In addition, a Pirani gauge (*STINGER*, *CVM211 series*) was used to monitor the pressure in the experimental chamber. The real-time temperature measurement of the solidifying sample was performed using an optical pyrometer (*Chino*, *IR-CAS series*; measurement range 600–3000 °C). The operating wavelength of pyrometer is 900 nm. Appropriate emissivity corrections have been applied to maintain uniformity in the measured values of temperature over the design range. The pyrometer is integrated with a close-up lens that enables focusing on the surface of the molten droplet to make sure that, to the maximum possible extent, the pyrometer looks at the surface of the droplet and the radiations from the off-focal points do not fall on the detector.

## Materials and methods

In order to ensure consistency across the various experiments performed, starting material (Mg_2_SiO_4_ with 99.9% purity, *Sigma Aldrich, USA*) was prepared by carefully weighing its powder sample using a Mettler Toledo digital balance. The weighed powder was placed on a copper hearth, and exposed to laser radiation. The radiation melted the powder and subsequently, spherical samples were formed under the high surface tension of the melt. The sizes of the thus prepared samples were measured and were found to be in the range 2 ± 0.3 mm. Vacuum-tweezers were used for handling these solid samples (spherules) and to place (or lift) them on the nozzle of the levitator before the start (or end) of any given experiment. The stability of the levitated molten samples and constancy of initial temperature in the experiments were maintained by carefully adjusting both the laser power as well as the gas flow rate.

For all the experiments reported in this article, the levitation was achieved using argon gas. Stable levitation of the molten droplet became a bigger challenge while performing such container-less experiments under sub-atmospheric conditions. In this direction, to ensure stable levitation of the spherule, a leak was introduced in the chamber by fitting one of the ports (diametrically opposite to the suction port) with a nozzle valve. Initially, the vacuum pump was run at its full capacity while the nozzle valve remained fully open. This was almost similar to levitation under atmospheric conditions. Thereafter, the nozzle valve was closed slowly while controlling the incoming gas flow and the laser power. The gas inlet was controlled so as to maintain a balance between the gas being pumped out and the gas being pumped in. Power of the CO_2_ laser was regularly controlled in order to maintain the temperature of the molten/crystallizing sample at the desired levels. Temperature of the sample was continuously monitored using an optical pyrometer.

Experiments were conducted under atmospheric condition and sub-atmospheric conditions (50 mbar). The initial temperature of the molten droplet was maintained at ~ 2400 K in all the experiments. Three different cooling rates (100, 200 and 400 K/s) were set to compare the results. The time vs. temperature curves were recorded for all the experiments. Images of the crystallizing spherule at the corresponding times were also recorded. All the diagnostics, viz. pyrometer and high-speed camera was synchronised with the time of the clock of HTCNL system to establish a one-on-one correlation.

## Results and discussion

Two sets of experiments were conducted—one, under atmospheric pressure and the other under sub-atmospheric conditions (pressure of 50 mBar). The processes of crystallisation and the associated phenomenon of recalescence were observed and recorded in terms of temperature vs. time data as well as in the form of time-stamped images recorded using high speed camera. A detailed discussion on the effect of pressure conditions, cooling rate and the evolution of the surface features has been presented in this section. A vis-à-vis comparison based on the temperature–time history and evolution of surface morphologies in the two cases has also been presented.

### Comparison based on temperature–time-history

**Figure 2:**
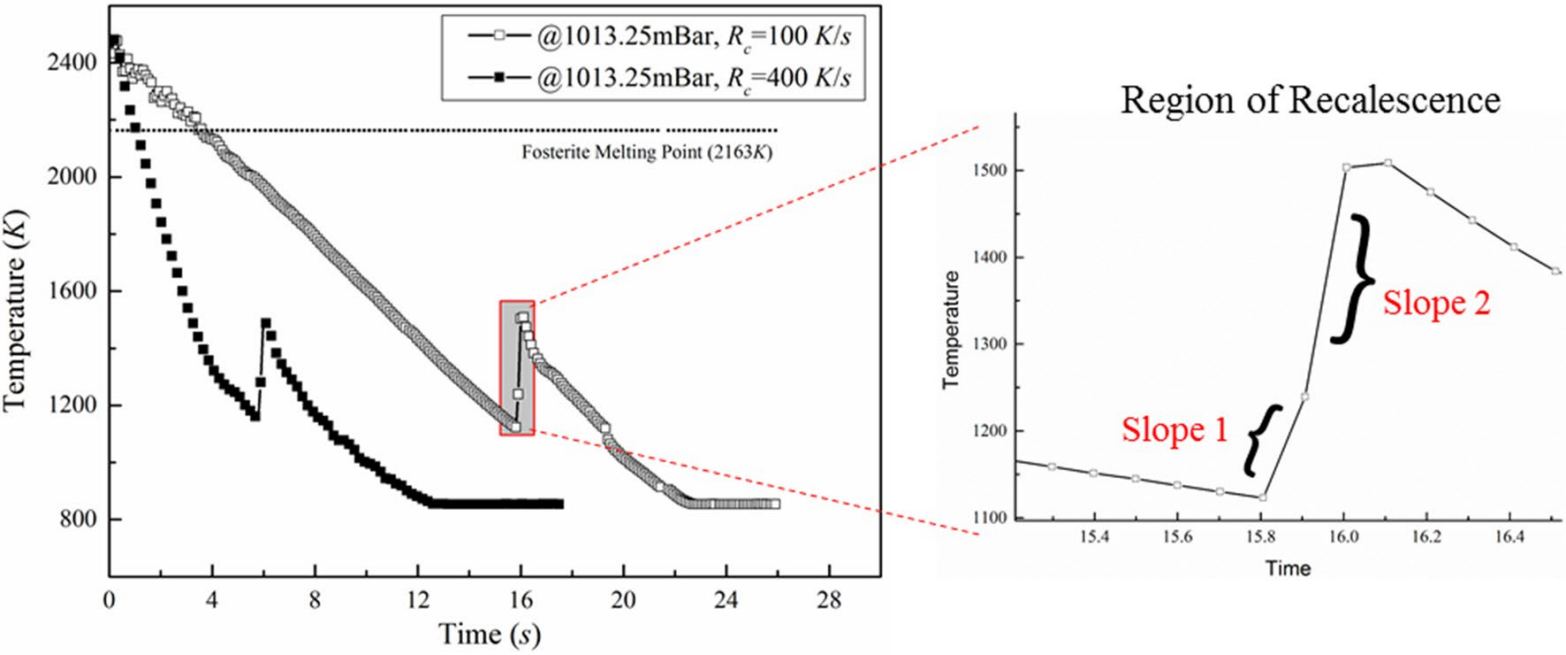
Temperature–time history of molten samples cooled at 100 K/s and 400 K/s.

In order to ensure complete melting of the starting material, solid spherules (diameter ≈ 2 mm) of forsterite composition (Mg_2_SiO_4_) were heated to a temperature of ~ 2400 K, which is ~ 250 K higher than its melting point (*T*_M_ = 2163 K). These conditions of almost constant temperature of the molten droplet were ascertained through the pyrometer readings over time. Once in molten state, the droplet was let to cool at different rates. Interestingly, all the samples were observed to have hypercooling before the onset of the crystallization process, irrespective of the cooling rate applied, as may be seen from Fig. 2. The solidifying samples were observed to undergo recalescence, a phenomenon which is associated with the release of latent heat of crystallization, which subsequently leads to a sudden increase in the temperature of the crystallizing sample volume. Signatures of the recalescence phenomenon are directly evident from the cooling curves shown in Fig. 2 for any given cooling rate that the molten droplet is subjected to.

The cooling curves of two identical forsterite samples, which were let to cool down at 100 and 400 K/s, have been shown in Fig. 2. It can be seen that, for the cooling rate of 400 K/s, which is four times as high as compared to 100 K/s, the cooling time is scaled more or less by the same factor. At relatively lower cooling rate, the temperature gradient between the melt’s outer surface and its center is expected to be weak as the molten volume has enough time to homogenize the temperature distribution. Thus, the occurrence of any sort of instabilities within the melt due to strong non-uniform temperature gradients can reasonably be ruled out and hence, more or less uniform crystallisation of the solidifying droplet may be expected.

Suppression of thermal instabilities within the molten volume can also lead to the realization of relatively higher values of undercooling as compared to the case when the cooling rate is relatively high. However, as shown in Fig. 2, the observed difference is quite less (~ 50 K); which accounts for just 5% of undercooling that the samples undergo before crystallization. This is clearly observed from the averaged values of the undercooled temperature and degree of undercooling presented in Table 1 for different cooling rates (the averaging has been done after considering data from sufficient number of experiments carried out under the same conditions). Crystallization is seen to occur from hypercooled melts (the hypercooled limit for forsterite is 425 K^34^), which is corroborated from the data given in Table 1. However, the effect of varying cooling rates on the crystallization phenomenon is observed during the recalescence process when recorded by the high-speed camera.

**Table 1:** Averaged undercooled temperature and degree of undercooling for various cooling rates under atmospheric conditions.

Cooling rate (K/s)	Undercooled temperature (K)	Degree of undercooling (K)
100	1180.7	982.29
200	1124.32	1038.68
400	1132.62	1030.37

The zoomed-in section of Fig. 2 shows the temperature–time history of the sample while it undergoes crystallization. The sampling rate of the pyrometer (10 Hz) was fast enough to capture at least three data points within the recalescence region. These three data points give two different slopes which have been considered while comparing the cases of different cooling rates. The data from the pyrometer has been used to estimate the rate of change of surface temperature with time. As the cooling process starts, the fall in temperature (before the point of recalescence) depends only on the rate of cooling that the sample is subjected to. However, during the process of recalescence, the latent heat of crystallisation is released, which leads to a sudden temperature gradient between the surface of the molten droplet and the surrounding atmosphere. Therefore, the rate of change of temperature is expected to depend on the inner dynamics of the melt as well, along with the cooling rate. However, within the range of uncertainties involved, it is safe to state that the gradient is independent of the cooling rate employed.

**Figure 3:**
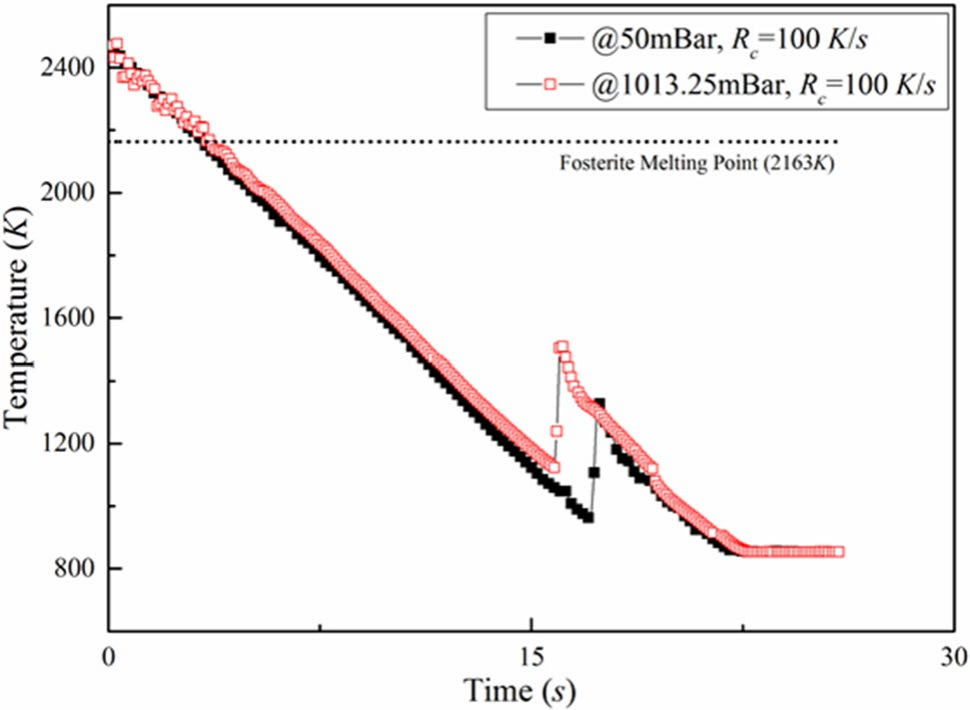
Cooling curves of the molten droplets under atmospheric and sub-atmospheric (50 mBar) conditions (Cooling rate: 100 K/s).

Figure 3 presents the temperature–time history of two separate, yet of identical size and composition, forsterite samples subjected to a cooling rate of 100 K/s but at two different pressure conditions, viz. atmospheric pressure and at a sub-atmospheric pressure of 50 mBar. It is clearly seen that there is no difference in the pre-recalescence phase, as both the samples are subjected to the same rate of cooling. However, the experiments conducted under atmospheric conditions revealed that the solidifying sample approaches the limit of undercooling earlier than the molten droplet levitated under sub-atmospheric condition. The onset of nucleation can clearly be seen to have delayed for the sample that is subjected to sub-atmospheric conditions.

The observed delay in the onset of nucleation under sub-atmospheric conditions can possibly be attributed to the suppression of convective thermal instabilities within the volume of the molten droplet. Under normal atmospheric conditions, along with radiative heat transfer being the dominant mode of energy transfer from the molten droplet, the presence of temperature gradients between the sample surface and the surrounding ambient medium leads to convective plumes and hence, forced convective cooling also plays a prominent role during the solidification process. Convective instabilities, set-up within the volume of the molten droplet, have been shown to be one of the causes for early onset of nucleation. For instance, Nagashima et al.^16^ performed similar experiments under microgravity conditions and showed a significant increase in the level of undercooling that can be achieved before the sample starts to nucleate under such reduced gravity conditions, as compared to relatively low levels of undercooling under normal atmospheric conditions. The authors attributed the realization of higher levels of undercooling under microgravity conditions to the possible suppression of convective instabilities. The observations made in the present work (Fig. 3) are in line with that reported by Nagashima et al.^16^. However, the importance of the present work gets highlighted by the fact that, compared to performing such experiments under microgravity conditions, the lab-based levitation experiments carried out under sub-atmospheric pressure conditions is one of the cost-effective means of achieving more or less similar results as far as levels of undercooling is concerned. In the present work, even under vacuum conditions, the requirement of container-less conditions has been achieved (contrary to the work of Nagashima et al.^16^ wherein the sample was held using a thin PtRh wire and/or a pair of PtRh rods), which, in turn, has allowed to delay the onset of nucleation and achieve relatively higher levels of undercooling in comparison with the container-less experiments performed under normal pressure conditions (Fig. 3).

**Figure 4:**
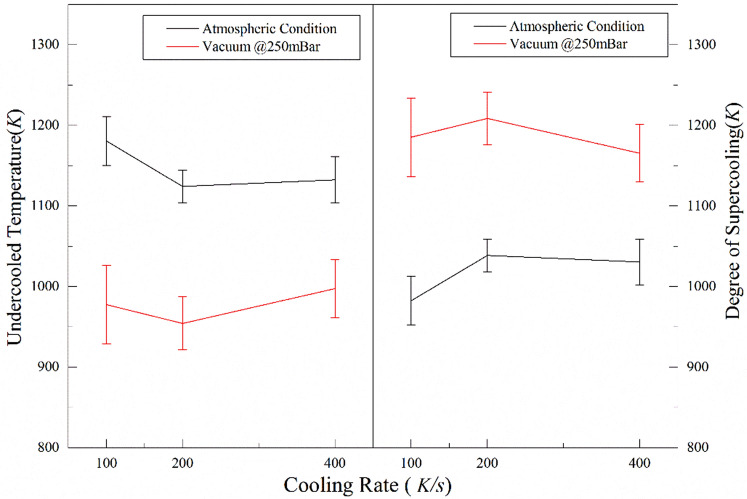
Comparison of undercooled temperature and the degree of supercooling for different cooling rates under atmospheric and sub-atmospheric conditions.

Suppression of convective currents under sub-atmospheric conditions (50 mBar) prolongs the sustenance of the molten state of the sample and delays the onset of nucleation. This trend was seen irrespective of the cooling rate employed in the experiments. For better clarity, a direct comparison of the undercooled temperatures (as well as the degree of supercooling achieved) under atmospheric and sub-atmospheric conditions, as a function of cooling rates, has been shown in Fig. 4. While there is a stark difference in the values of undercooled temperatures achieved under atmospheric and sub-atmospheric conditions for any given cooling rate, the temperature values are almost insensitive to the variations in the cooling rates employed. For instance, at a cooling rate of 100 K/s*,* the undercooled temperature to which the levitated sample could be sustained in molten form before the onset of nucleation at 50 mBar is ≈ 975 K (± 50 K) [corresponding degree of supercooling ≈1185 K (± 50 K)]. On the other hand, normal atmospheric pressure conditions offered an undercooled temperature of ≈ 1175 K ± 30 K, which amounts to the degree of supercooling of ≈ 980 K ± 30 K, which is significantly lesser than that achieved under sub-atmospheric conditions.

In the context of solidification from high temperature melts, phenomenon of recalescence plays an important role. It pertains to the increase in the local temperature of the solidifying melt due to the release of latent heat of crystallization. Under certain conditions, the increase in the local temperature can achieve those levels that are high enough to re-melt some portion of the solidifying front within the volume of the sample, which in turn, leads to local instabilities in the crystallizing front. These observations, which find support in some of the earlier works reported in the literature^35,36^, highlight the importance of the role played by the recalescence phenomenon and how quickly the latent heat of crystallization may be removed from the volume of the solidifying molten sample. In the context of the present work, the size of the molten droplet is extremely small (≈ 2 mm in diameter). Owing to the small size of the sample, the surface-to-volume ratio gets increased, and hence under normal atmospheric conditions, the latent heat of crystallization gets removed primarily through thermal radiation from the surface of the molten droplet, along with some contribution of convective cooling as the molten sample interacts with its surroundings. In contrast, sub-atmospheric/vacuum conditions are expected to offer quicker release of latent heat of crystallization as the heat dissipation would be primarily driven (controlled) by thermal radiation from the volume of the molten droplet.

In the experiments reported, the time history of temperature of the molten/crystallizing droplet was recorded using an optical pyrometer. At the instant of onset of nucleation, the released latent heat of crystallization leads to an increase in the surface temperature of the levitated sample. Thus, the slope of the segment of temperature vs. time curve that corresponds to the recalescence phase of the cooling curve can be considered to be an indicative of the rate at which the latent heat of crystallization gets dissipated from the system. This, in turn, would affect the rate at which the overall volume of the molten droplet would cool down. From the temperature–time history, the slope of the recalescence curve was determined. The average slope of the recalescence curve under sub-atmospheric conditions was found to be ≈ 1420 K/s whereas for experiments performed at standard atmospheric conditions, the average slope was ≈ 1217 K/s. In addition to the difference in the slopes of the recalescence curves, reasonable difference in the levels of temperature rise due to the phenomenon of recalescence were observed in atmospheric (≈ 350 K) and sub-atmospheric conditions (≈ 450 K). It is to be noted here that even though there is a higher degree of recalescence in the sub-atmospheric case, the melt appears to crystallize within the same time duration as in the case of atmospheric case (Fig. 3) giving rise to relatively higher slope in the recalescence curve. This observation also implies that the melt undergoes faster crystallization process under sub-atmospheric conditions and hence, one can expect finer morphological features in the crystallized sample. Direct experimental evidence of this inference is provided in the following sections.

### Solidification textures under atmospheric and sub-atmospheric conditions

**Figure 5:**
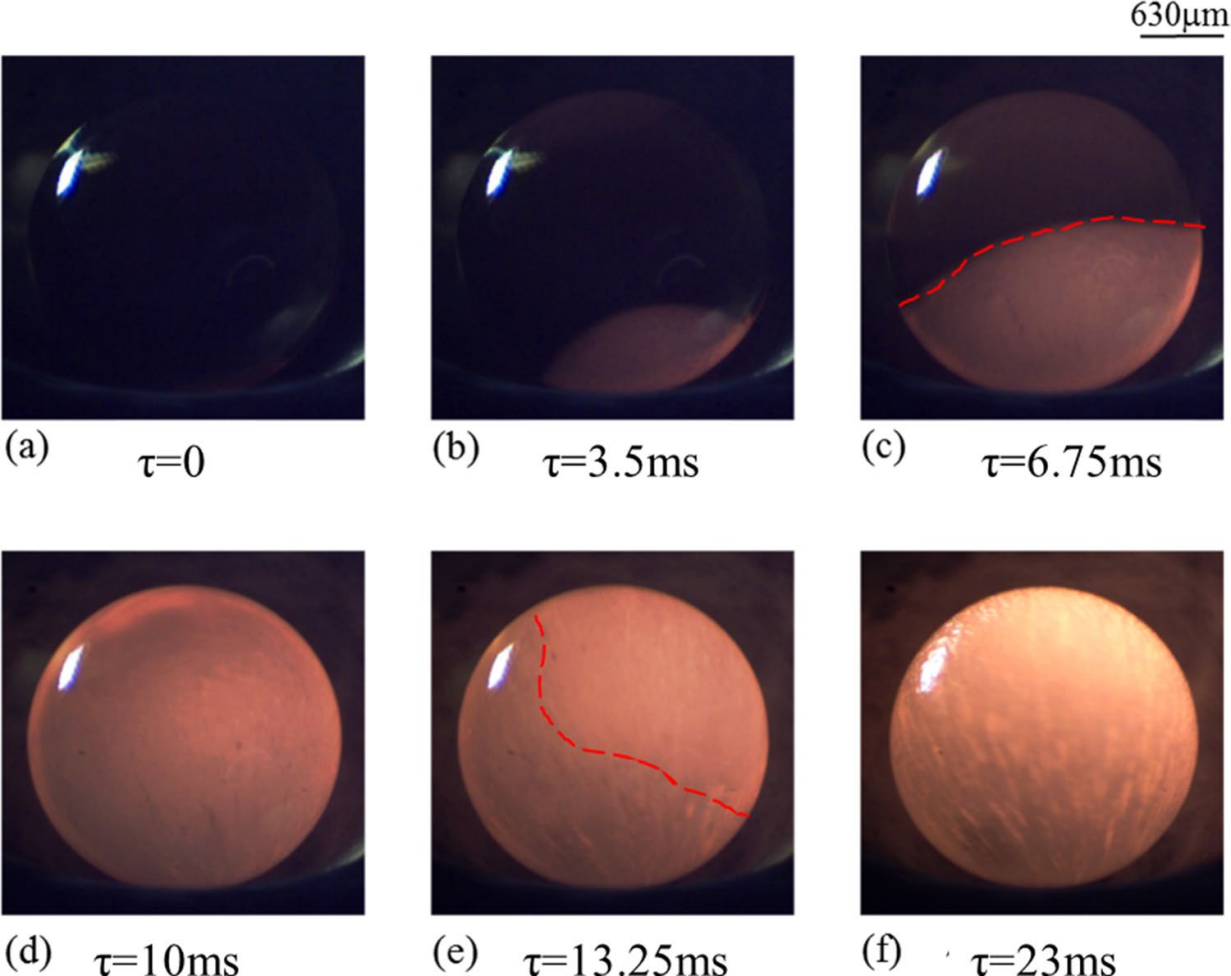
Time-sequenced images of solidification of molten sample subjected to a cooling rate of 100 K/s under atmospheric conditions. (For better visualization, see movie of the process supplied as Supplementary Video 1).

**Figure 6:**
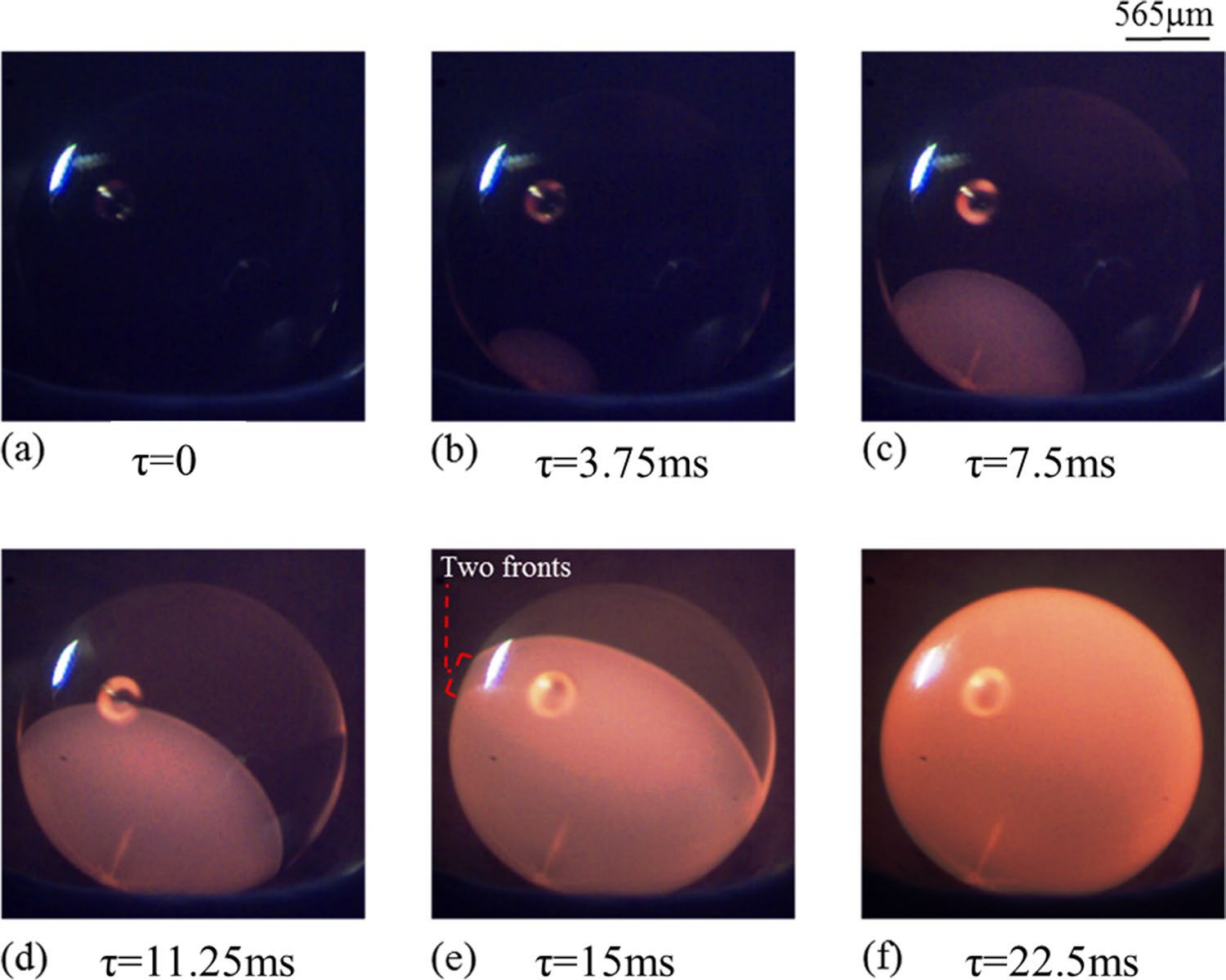
Time-sequenced images of solidification of molten sample subjected to a cooling rate of 400 K/s under atmospheric conditions. (For better visualization, see movie of the process supplied as supplementary Video 2.

The plausible effect of varying pressure conditions on the textures of the crystallizing spherules have been discussed in the present section. The data, recorded in the form of time-lapsed 2D images of the solidifying sample using a high-speed camera, pertains to two different cooling rates (100 and 400 K/s). Figures 5 and 6 respectively show the transient evolution of the process of crystallization as the levitated molten droplet is subjected to cooling rate of 100 K/s (Fig. 5) and 400 K/s (Fig. 6) under atmospheric conditions. Note that *τ* = 0 corresponds to the time instant just before the nucleation process starts. Thus, the first image shown in both the figures (recorded at *τ* = 0) corresponds to the maximum level of undercooling achieved in the respective cases.

The onset of nucleation is evident in the form of localized increase in the brightness of the sample as a result of release of latent heat of crystallization. Second image in the first row of Figs. 5 and 6 demonstrates this phenomenon. With the passage of time, as the solidification front moves ahead, the release of latent heat of crystallization illuminates the complete volume of the solidifying spherule. The data presented in Figs. 5 and 6 clearly show that the initially molten droplet gets crystallized in a very short span of time (≈ 23 ms) even under normal pressure conditions. These observations support the theory of rapid crystallization of chondrules^16,37^.

Comparing the time-lapsed snapshots of the crystallization processes shown in Figs. 5 and 6, it becomes evident that the solidification textures are quite different. In contrast to the case of relatively higher cooling rate (400 K/s) that results in a clear rim-like solidification front, the spherule subjected to the cooling rate of 100 K/s shows a diffused rim-like front (Fig. 5e). In the case when the surface of the spherule is subjected to relatively higher cooling rate (400 K/s), sharp temperature gradient between the center of the droplet and its surface is to be expected with the central core region of the droplet being at relatively higher temperature (also shown by Miura et al.^24^). The preferential rapid cooling of the surface of the droplet leads to a sharp rim-like solidification front, as to be clearly seen from the images shown in Fig. 6. In contrast, at relatively lower cooling rate (100 K/s), the time-stamped snapshots in Fig. 5 reveal the propagation of a diffused rim-like front on the surface of the undercooled molten droplet as well as distinct signatures of crystallization from the central core of the droplet towards its surface. Initiation of crystallization from the centre of the droplet, along with its surface, can be attributed to the fact that surface of the molten droplet is subjected to relatively lower cooling rate (100 K/s). As the surface cooling is slow, the undercooled molten droplet has sufficient time to achieve near homogenous distribution of temperature over its entire volume, which allows almost simultaneous crystallization of the surface of the droplet as well as inside of the molten volume. This is evident from Fig. 5e,f wherein, along with the appearance of a diffused rim, one can clearly observe radial growth of structures from the point of initiation of the nucleation process. These real time experimental observations find support in the work of Miura et al.^24^ wherein the authors reported the results of numerical simulations to study the crystallization of hypercooled melt under heterogenous nucleating conditions. A quasi-planar growth interface with solid growing radially from the seeding point was reported. However, in the present case, radial growth along with the formation of a diffused-rim like structures is observed.

In contrast to the results presented in Fig. 5, images in Fig. 6 predominantly indicate towards crystallization taking place along the outer surface of the droplet without any distinct morphological features inside its volume. Careful observation of the images shown in Figs. 5 and 6 reveals that, at a higher cooling rate, the solidification front maintains the curvature of the rim-like structure right from the onset of recalescence to the end. Whereas, in the case of lower cooling rate, the semi-circular shape of the front tends to become flat as the time elapses.

In order to assess the effect of cooling rate on crystallization of highly undercooled molten droplets, the crystallized spherules were subjected to SEM characterization. The electron micrographs of the surface features of the crystallised samples showed the presence of large-sized surface textures in the case of lower level of cooling rates, whereas, surface features were found to be relatively very small when the molten droplet was subjected to higher cooling rate at 400 K/s. These observations are along the expected lines and may be explained on the basis of the availability of cooling time for the highly undercooled molten droplet^38–40^.

**Figure 7:**
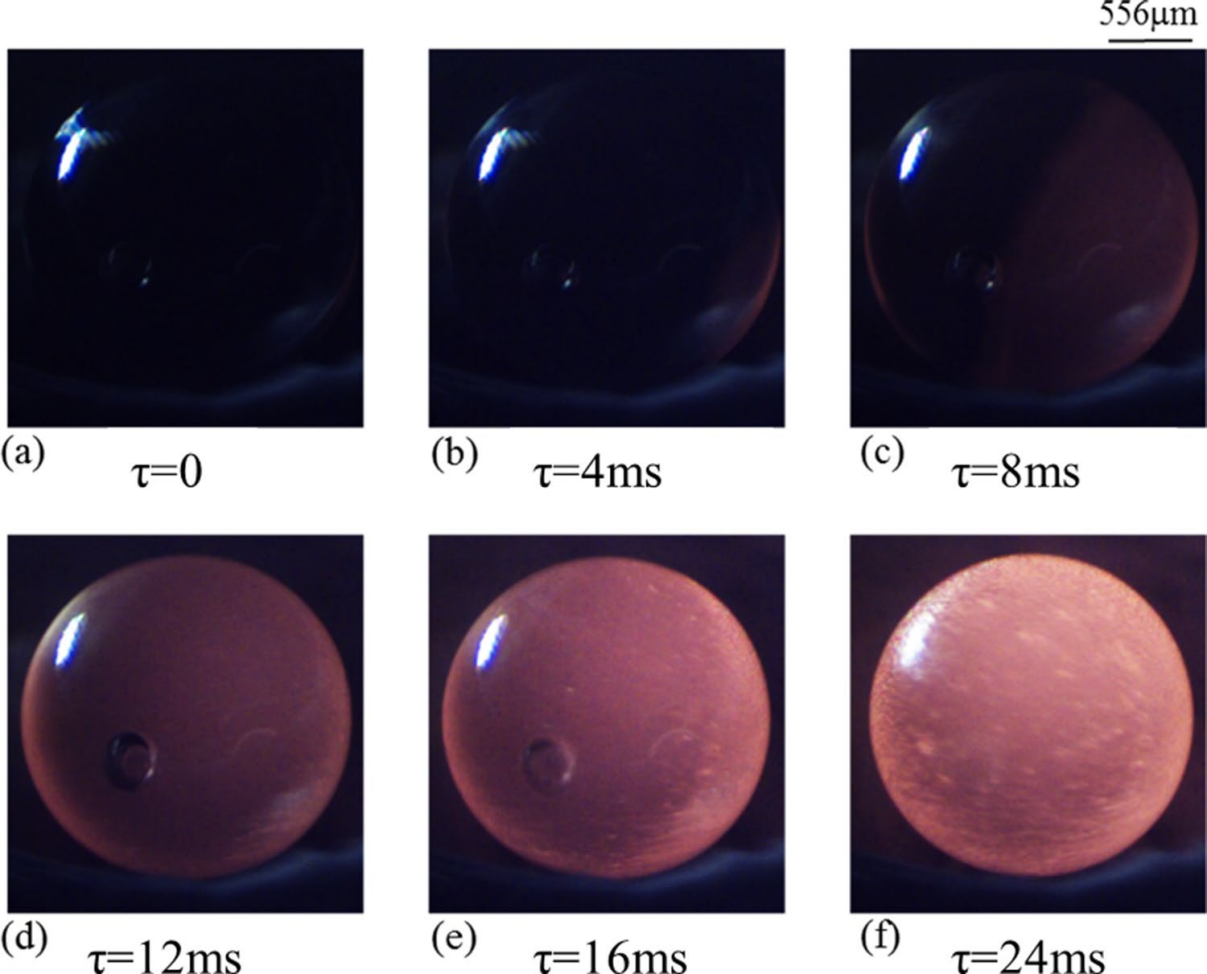
Time-stamped images of solidification of molten sample subjected to a cooling rate of 100 K/s under 50 mBar sub-atmospheric conditions. (For better visualization, see movie of the process supplied as Supplementary Video 3).

**Figure 8:**
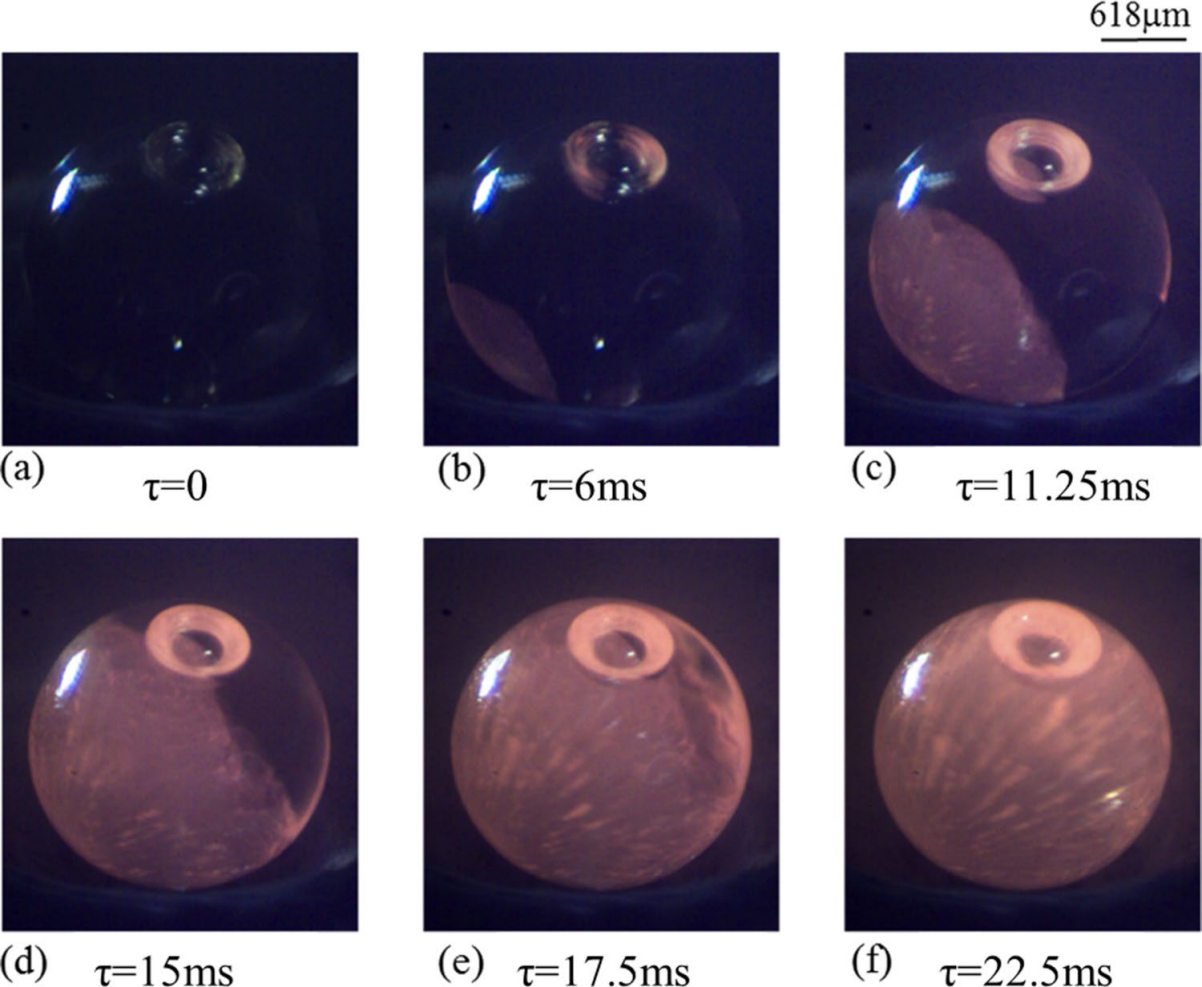
Time-stamped stamped images of solidification of molten sample subjected to a cooling rate of 400 K/s under 50 mBar sub-atmospheric conditions. (For better visualization, see movie of the process supplied as Supplementary Video 4).

Real time observations of the crystallization phenomenon achieved under sub-atmospheric conditions have now been presented to elucidate the plausible impact of reduced pressure conditions on morphological textures. In order to ensure a direct comparison, all other experimental parameters, for instance, sample size and its composition, cooling rates employed etc. have been kept identical to those employed in the standard atmospheric pressure conditions. Figures 7 and 8 show the time-lapsed images of the crystallization phenomena as the highly undercooled molten spherules is subjected to cooling rates of 100 K/s (Fig. 7) and 400 K/s (Fig. 8) under sub-atmospheric pressure conditions.

As discussed in “Comparison based on temperature-time-history”, compared to normal pressure conditions, the molten spherule could be undercooled to relatively much higher levels of supercooling under sub-atmospheric conditions before the onset of nucleation. Furthermore, the dissipation of the latent heat of crystallization during the recalescence phase of the process was seen to take place at a much faster rate under sub-atmospheric conditions. This comparison was made using the slope of the temperature vs. time curve corresponding to the recalescence phase of the process. Faster removal of the latent heat of crystallization under sub-atmospheric conditions leads to rapid cooling of the entire volume of the molten droplet (as the heat dissipation phenomenon is primarily driven by volumetric thermal radiation) as against the surface cooling of the high temperature molten droplet that takes place through the mechanism of thermal radiation and convective heat transfer under normal pressure conditions. Thus, irrespective of the magnitude of the externally employed cooling rate, the undercooled molten droplet, after its crystallization, is expected to have small length scale morphological textures under sub-atmospheric conditions, in comparison to those achieved under normal pressure conditions.

Furthermore, for any given cooling rate, under reduced pressure conditions, the molten droplet is expected to crystallize not only from the outer surface but also from its inside. It is to be noted here that almost simultaneous initiation of the crystallization process from the surface as well as from inside of the molten droplet was observed only in those experiments wherein the undercooled melt was subjected to relatively lower level of cooling rate (100 K/s) under normal atmospheric conditions (Fig. 5) while at higher cooling rate of 400 K/s, the crystallization phenomenon was seen to take place primarily on the outer surface of the spherule before the complete volume of the molten sample got crystallized (Fig. 6). In contrast, the experimental images shown in Figs. 7 and 8 clearly indicate towards volumetric crystallization of the undercooled molten spherule. Even at relatively higher cooling rate (400 K/s), the crystallization fronts initiating from inside of the volume of the molten spherule can be seen to be closely following the surface crystallization front, as depicted in Fig. 8. These features, recorded under sub-atmospheric conditions and at an externally employed cooling rate of 400 K/s, are strikingly different from those observed under normal pressure conditions, wherein only surface crystallization front was observed (shown earlier in Fig. 6).

Comparing the snapshots shown in Fig. 7 (sub-atmospheric conditions) and 6 (atmospheric conditions), one observes relatively uniformly distributed stripes (crystallization fronts inside the volume of the molten spherule) of smaller length scales at any given comparable time instant (say, around τ ≈ 22.5 ms) under sub-atmospheric conditions vis-à-vis those obtained under normal pressure conditions. Thus, even if the externally applied cooling rate is same, molten spherule under sub-atmospheric conditions observes faster cooling that is quite uniform over its entire volume in contrast to that under normal pressure conditions wherein the surface cooling of the droplet dominates. The observed features indicate that the molten spherules crystallizing under sub-atmospheric conditions would have an almost uniform temperature profile as one moves from its center towards its periphery. This is in contrast to the case of the undercooled melt solidifying under normal pressure conditions wherein the core of the droplet remains at significantly higher temperature than its surface. From Fig. 8e, the surface front is observed to have concluded earlier than the volumetric front, suggesting that although both the fronts initiate and grow simultaneously, yet the volumetric crystallization takes a little longer to conclude. A discussion on the same supported by the required experimental observations would be presented in the next section.

**Figure 9:**
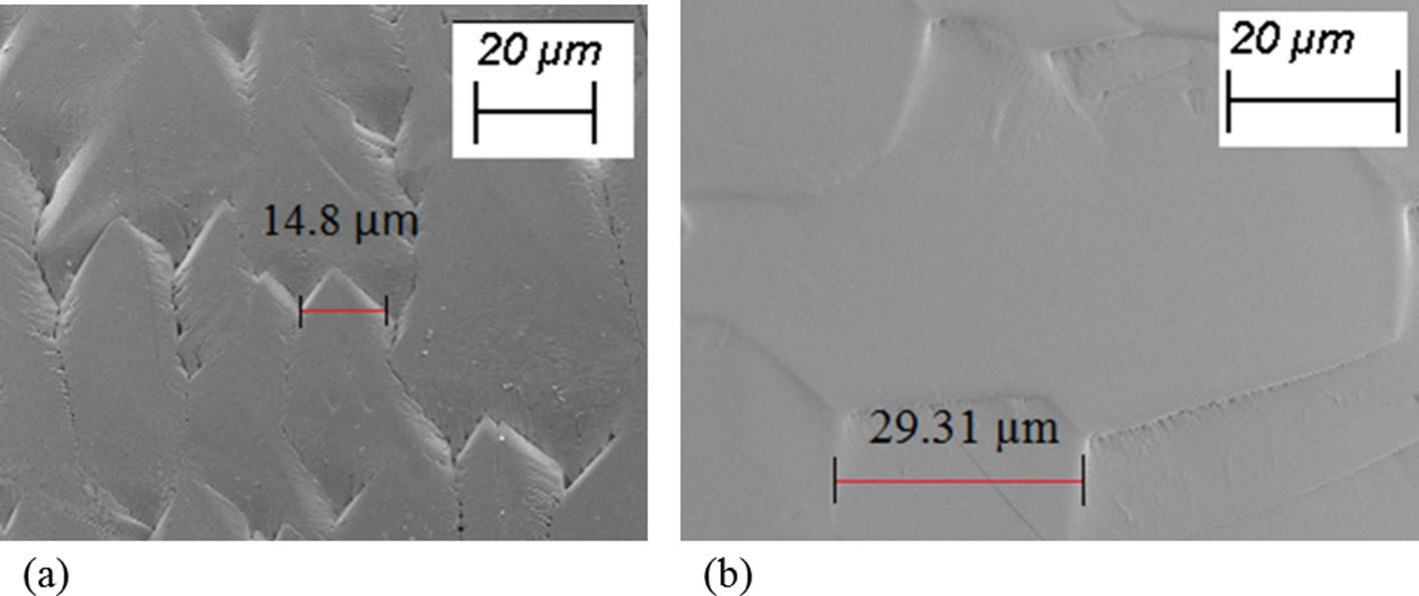
Comparison of the surface textures of forsterite melts crystallized with a cooling rate of 100 K/s under sub-atmospheric conditions (**a**) and atmospheric conditions (**b**).

Figure 9a,b show the electron micrographs of the surface of the spherules crystallized under sub-atmospheric and atmospheric conditions when cooled at a rate of 100 K/s. It may be clearly seen that due to faster rate of cooling post-recalescence, the features developed on the surface of the solidified spherule under sub-atmospheric conditions are smaller compared to the features observed on its counterpart cooled under atmospheric condition. This corroborates the results reported by researchers that faster cooling and higher extent of undercooling lead to finer surface structures^40,41^. It is to be highlighted here that the images shown in Fig. 9 are representative and are in proper agreement with the statistical trend observed from many such images taken at various locations of the sample surfaces. Similar results were also obtained in the case of 400 K/s.

### Reproduction of double structure of rim and dendritic under sub-atmospheric conditions

Based on 2D numerical simulations, Miura et al.^24^ discussed the possibility and growth conditions of a unique double structure comprising of rim and dendrites from highly supercooled melt droplets subjected to heterogenous nucleation. Such unique morphological feature holds importance in the context of chondrule crystallization, as some of the naturally occurring chondrule samples have revealed similar type of double structure^42^. With this as the motivation, select experiments were performed under sub-atmospheric conditions to reproduce such rim + dendritic features from the container-less solidification of highly undercooled molten droplets of forsterite composition. The stark differences in the study reported here and that by Miura et al.^24^ include; (1) Miura et al.^24^ performed numerical simulations to reproduce such a structure under normal pressure conditions, however, the present results correspond to non-contact experiments under sub-atmospheric conditions, (2) Nucleation was heterogeneously initiated in Ref.^24^, whereas in the present experimental work, nucleation has been initiated under completely non-contact conditions subjecting the levitated molten droplet to high level of undercooling, and (3) the external cooling rate employed in the present work is almost two-to-three times lower than that employed in Ref.^24^. In view of the fact that the present experiments have been conducted under reduced pressure conditions and that too under non-contact conditions, as opposed to the numerical study of Miura et al.^24^, the present experimental observations can be expected to explain the formation mechanism of such a unique double structure in naturally encountered chondrule samples in a more realistic term.

**Figure 10:**
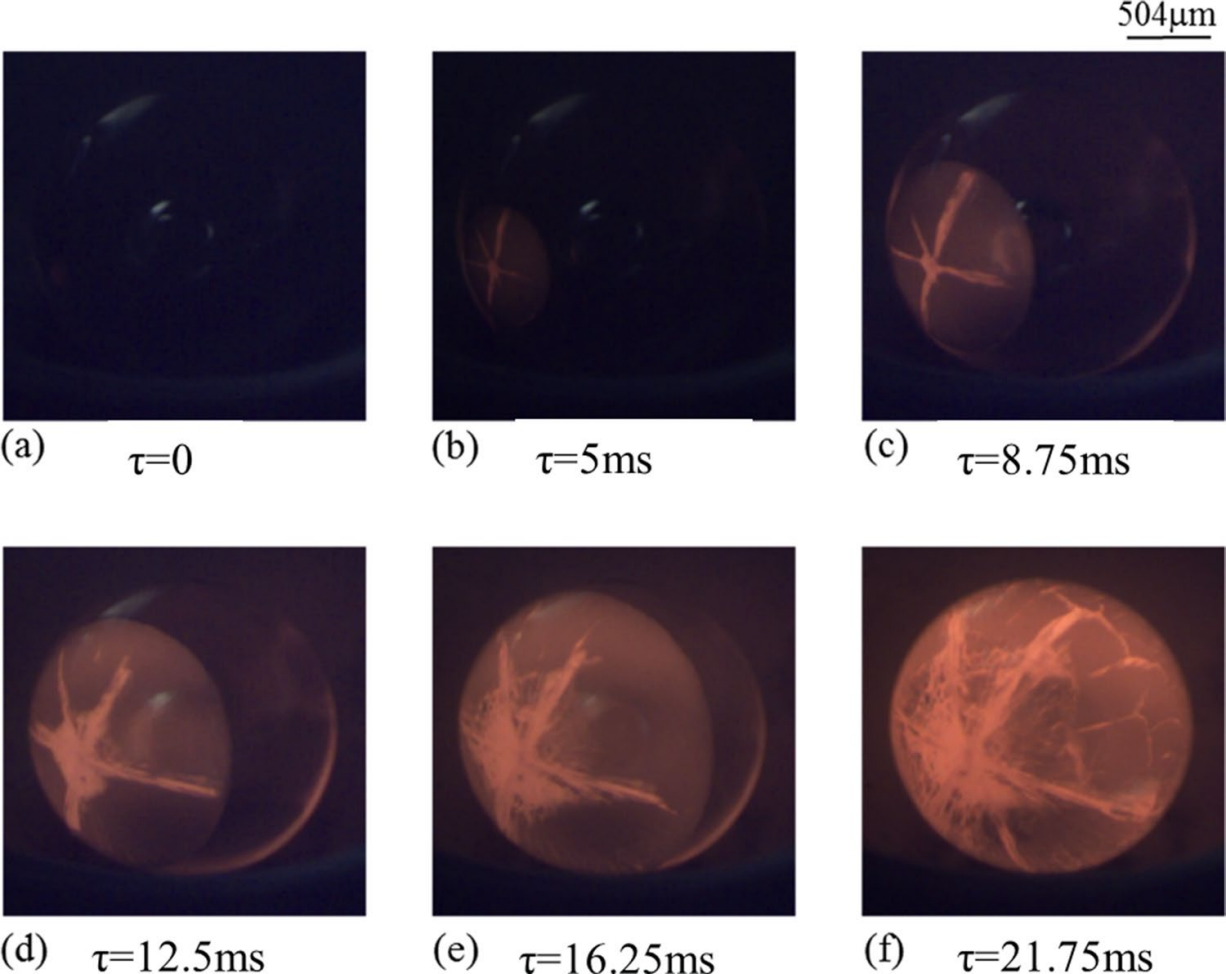
Time-stamped stamped images of solidification of molten sample subjected to a cooling rate of 400 K/s under 50 mBar sub-atmospheric conditions. Evolution of double structure of Rim + Dendrites can be clearly seen from the image sequence shown. (For better visualization, see movie of the process supplied as Supplementary Video 5).

Figure 10 shows the time-lapsed images of crystallization process of levitated and highly undercooled forsterite melt droplet under sub-atmospheric conditions (system pressure equal to 50 mBar). The non-contact conditions ensured a high degree of undercooling before the onset of nucleation. The undercooling achieved in these experiments was ≈ 975 K (± 50 K). With reference to the images shown, the onset of nucleation can be seen to take place somewhere between *τ* = 0 and *τ* = 5 ms and the signature of the start of crystallization process can be seen in the form of localized increase in the brightness in the image recorded at *τ* = 5 ms (due to the release of latent heat of crystallization). Starting from *τ* = 0, with the passage of time (Fig. 10a–f), dendritic arms and rim both are distinctly visible.

During the initial time instant (Fig. 10b), both the rim as well as dendritic fronts can be seen to have grown to the same extent and the tip of the dendritic arm (formed due to volumetric cooling) just touches the perimeter of the forward moving rim (formed primarily due to solidification of the surface). Simultaneous development of both these structures during the initial phase of the crystallization process suggests that both the features grow at the same rate. However, the path of travel is different for each of them with the rim growing on the surface of the solidifying droplet, while the dendrite grows through the center of the droplet towards the outer surface. Over the period of time, both grow at a competitive pace as can be seen in Fig. 10d wherein one of the arms of the dendrite is still able to match the speed of growth of rim. But as the time progresses, the dendritic growth is seen to have slowed down and the distance between the rim and dendrite is observed to increasing, Fig. 10e. The growth of dendrites lags the faster moving growth of the rim structure along the surface of the solidifying droplet.

The observed trend may be explained as follows: The rim grows along the surface and its rate is controlled by the temperature gradient between the surface and the ambient, which, in turn, depends on the externally applied cooling rate. Since the cooling rate applied is constant, the growth rate of the rim is also constant. However, the growth of dendrite results from cooling over the volume, which means that it will always depend on the temperature gradient between the surface and interior of the molten droplet. Under any given condition, there will be a gradient (in temperature) existing between the center and the surface of the solidifying molten droplet. Compared to the normal atmospheric pressure conditions, this spatial gradient of temperature will impede the growth of the dendrite, which starts form the surface and moves towards the center. Although as per the discussion in the preceding sections, the strength of such temperature gradients is less in sub-atmospheric conditions, still it is strong enough to affect the dendritic growth. Though slowed down, yet the dendritic growth continues and it reaches the center where the temperature is highest. The difference between this temperature at the center of the droplet and the surface temperature ultimately controls the speed of dendritic growth. As the dendrites cross the center, their growth speed picks up as the temperature starts decreasing from the center to the surface and hence, the resistance also begins to decrease. The rim growth gets completed by the time the dendritic growth reaches the other side of the sample surface. The completion of the recalescence process can be seen in the image shown in Fig. 10f where all the dendritic arms are observed to have spread all over the volume of the solidifying spherule.

Importance of results presented in Fig. 10 can be realized by the fact that, to the best of the knowledge of the authors, this is one of the very first attempts to experimentally reproduce such a chondrule-like texture of double structure under sub-atmospheric conditions. Previous attempts reported in the literature are based on numerical simulations (Miura et al.^24^) and that too these simulations did not consider the crystallization phenomenon under reduced pressure conditions and also relied on heterogenous nucleation to start the crystallization process. In contrast, as a definite improvement towards realizing more realistic conditions that are believed to be associated with the mechanisms of natural chondrule textures, the present experiments have been performed under reduced pressure conditions and that too under non-contact conditions. In view of these inherent differences in the two approaches, the results presented in Fig. 10 also suggest that the growth mechanism(s) of double structure (rim + dendrite) need not be unique to a given case, and may well be suited to explain different cases. Successful reproduction of double structure (rim + dendrite) under sub-atmospheric conditions and at a cooling rate that is about 2–3 times slower than that employed by Miura et al.^24^ supports this conclusion. Results further explain the plausible role of reduced pressure conditions (that lead to faster dissipation of the latent heat of crystallization through volumetric cooling of the molten droplet eventually resulting into much higher cooling rate compared to the externally employed rate) in reproducing similar textures that have been reported in the numerical works, albeit under standard ambient pressure conditions.

## Conclusions

Experiments, conducted under purely non-contact conditions, were performed to investigate the plausible effect of operating pressure conditions on the crystallization phenomena of high temperature silicate melt droplets. Molten spherules of forsterite composition were levitated using the principles of aerodynamic levitation. The choice of forsterite composition as the model material was based on its relevance in the area of planetary sciences, especially in the context of chondrules crystallization. A direct comparison of surface textures of the samples crystallized under sub-atmospheric conditions was made with those achieved under normal pressure conditions. The observed trends were explained on the basis of difference in the rates at which the latent heat of crystallization gets dissipated from the volume of the molten droplet under the varying pressure conditions. The phenomenon of onset of nucleation was seen to get delayed under sub-atmospheric conditions, which, in turn, allowed to achieve relatively higher levels of undercooling. Time-lapsed images of the crystallizing molten spherule, recorded using a high speed camera, showed that, irrespective of the externally employed cooling rate, the solidification phenomena under sub-atmospheric conditions results into small length scale morphological textures in comparison to those achieved under normal pressure conditions. Micrographs of the sample surfaces cooled under different pressure conditions showed different kind of morphologies that were commensurate with the processes that take place on the exterior and interior of the melt. Experiments were further conducted to successfully reproduce one of the important chondrule textures namely the double structure of rim + dendrites under sub-atmospheric conditions and that too under completely non-contact conditions. The formation conditions of this texture, as inferred from the present set of experiments, were compared with those reported in the numerical work by Miura et al.^24^. To the best of the knowledge of the authors, this experimental study is one of the first attempts to reproduce some of the important chondrule textures through container-less experiments under sub-atmospheric conditions. The correlation of surface textures with the operating conditions, as inferred from the present set of experiments, is believed to provide important insights on the formation conditions of some of the important chondrules textures.

## Supplementary information

Supplementary Information 1.

Supplementary Video 1.

Supplementary Video 2.

Supplementary Video 3.

Supplementary Video 4.

Supplementary Video 5.
