# Supplementary material for: Experiments to understand crystallization of levitated high temperature silicate melt droplets under low vacuum conditions
Source: Sci Rep. 2020 Dec 1;10:20910. doi: 10.1038/s41598-020-77965-4 (PMC7708509; doi:10.1038/s41598-020-77965-4)
Supplement: Supplementary file 1 — Supplementary Information 1. [file 41598_2020_77965_MOESM1_ESM.pdf]

Supplementary Material Information

Dear Editor
Scientific Reports-Nature

As per the guidelines of the journal, this document provides the details of the supplementary materials of our manuscript. We have referenced the supplementary videos supporting the observations reported at appropriate locations in the manuscript. The following are the details of the manuscript along with the complete list of the supplementary videos and their titles.

Manuscript	**Non-contact experiments to understand the effect of sub-atmospheric pressure conditions on crystallization of high temperature silicate melt droplets**
Authors	*Biswajit Mishra[1], Pratikkumar Manvar[1], Kaushik Choudhury[2], S. Karagadde[1] and Atul Srivastava[1*]*
Affiliations	*[1]Department of Mechanical Engineering, Indian Institute of Technology Bombay, Powai, Mumbai 400076, India*<br>*[2]School of Applied Sciences, KIIT, Bhubaneshwar, Odisha 751024, India*

List	Title
1. Supplementary Video S1:-	*Video_Figure5_supplementary material.mp4*
2. Supplementary Video S2:-	*Video_Figure6_supplementary material.mp4*
3. Supplementary Video S3:-	*Video_Figure7_supplementary material.mp4*
4. Supplementary Video S4:-	*Video_Figure8_supplementary material.mp4*
5. Supplementary Video S5:-	*Video_Figure10_supplementary material.mp4*

Each of the supplementary videos have been provided separately using their title names.

We would be happy to provide any further information in this regard.

Thank you and best Regards,

Atul Srivastava

Professor
Department of Mechanical Engineering
IIT Bombay, Powai, Mumbai 400076, India
Email: atulsr@iitb.ac.in
